# Balsam Poplar Buds: Extraction of Potential Phenolic Compounds with Polyethylene Glycol Aqueous Solution, Thermal Sterilization of Extracts and Challenges to Their Application in Topical Ocular Formulations

**DOI:** 10.3390/antiox11091771

**Published:** 2022-09-08

**Authors:** Monika Stanciauskaite, Mindaugas Marksa, Liudas Ivanauskas, Kristina Ramanauskiene

**Affiliations:** 1 Department of Clinical Pharmacy, Faculty of Pharmacy, Lithuanian University of Health Sciences, Sukileliai Avenue 13, LT-50162 Kaunas, Lithuania; 2 Department of Drug Chemistry, Faculty of Pharmacy, Lithuanian University of Health Sciences, Sukileliai Avenue 13, LT-50162 Kaunas, Lithuania; 3 Department Analytical & Toxicological Chemistry, Faculty of Pharmacy, Lithuanian University of Health Sciences, Sukileliai Avenue 13, LT-50162 Kaunas, Lithuania; mindaugas.marksa@lsmu.lt (M.M.); liudas.ivanauskas@lsmuni.lt (L.I.)

**Keywords:** balsam poplar buds, phenolic compounds, extract, ocular delivery

## Abstract

Phenolic compounds of natural origin have been valued for their beneficial effects on health since ancient times. During our study, we performed the extraction of phenolic compounds from balsam poplar buds using different concentrations of aqueous polyethylene glycol 400 solvents (10–30% PEG400). The aqueous 30% PEG400 extract showed the best phenolic yield. The stability of the extract during autoclave sterilization was evaluated. The extract remained stable under heat sterilization. Ophthalmic formulations are formed using different concentrations (8–15%) of poloxamer 407 (P407) together with hydroxypropyl methylcellulose (0.3%), sodium carboxymethyl cellulose (0.3%) or hyaluronic acid (0.1%). Physicochemical parameters of the formulations remained significantly unchanged after sterilization. Formulations based on 12% P407 exhibited properties characteristic of in situ gels, the gelation point of the formulations was close to the temperature of the cornea. After evaluating the amount of released compounds, it was found that, as the concentration of polymers increases, the amount of released compounds decreases. Formulations based on 15% P407 released the least biologically active compounds. Sterilized formulations remained stable for 30 days.

## 1. Introduction

Much attention is paid to ophthalmic preparations with biologically active compounds of natural origin, which are applied as anti-inflammatory and antioxidant agents [1,2]. This is due to the fact that in today’s society, with the fast pace of life and high flow of technology, eyes are subject to a lot of strain, fatigue and frequently develop dry eye syndrome and sensitivity, which leads them being more prone to infections [1,2]. Research shows a link between oxidative stress and ocular surface diseases [3,4] and one of the common solutions is the use of topical preventive eye products. Artificial tears are used to alleviate dry eye syndrome and corticosteroids are prescribed to reduce ocular surface inflammation [1,5,6]. Compounds of natural origin have a broadly applicable therapeutic effect, fewer side-effects and are more acceptable to the patient [5,7] than synthetic topical ocular preparations [7]. Some plant extracts have been used since ancient times, such as chamomile [8], calendula [9] and bilberry [10] extracts. Scientists are always looking for new sources of phenolic compounds for applications. One such source is poplar buds, which are often the plant precursor of propolis, and propolis itself has long been valued for its antioxidant, anti-inflammatory, antibacterial, anticancer and other therapeutic properties [11].

In Europe and Asia, the main plant sources of propolis are the bud exudates of Populus species and their hybrids [12,13]. In Lithuania, balsam poplar buds have been valued in folk medicine for their anti-inflammatory properties and have also been used to support respiratory and urinary tract functions. Preliminary research in Lithuania has identified salicin, *p*-coumaric, cinnamic, caffeic acids, the flavonoids pinocembrin, pinobanksin and galangin as the predominant phenolic compounds in balsam poplar buds [14].

The technology of manufacture is important in the development of ophthalmic formulations as they are intended for use on ocular tissue and must be sterile [15]. There are several technological processes that allow the production of sterile ophthalmic formulations: aseptic manufacturing, where the entire manufacturing process must be carried out under sterile conditions, or sterilization of the product at the final technological stage [16]. The sterilization methods available may be physical, such as filtration or autoclaving, or by chemical treatment [17]. Despite the various methods of sterilization of the formulation, it is important to select the most appropriate one according to the stability of the components of the formulation during the sterilization process. An inappropriate choice of sterilization method may adversely affect the stability of the ophthalmic formulation base or the active substance and may promote degradation of the compounds [18].

In order to apply plant material to the manufacture of ophthalmic preparations, it is important to isolate the active compounds from the raw material. At this stage, the selection of a suitable solvent that gives a good yield of biologically active compounds and is safe for the development of ophthalmic formulations is essential. A variety of solvents can be used for the extraction of the plant material, such as purified water, alcohols or organic solvents [19]. Organic and alcoholic solvents can achieve good extraction yields of bioactive compounds, but their application in pharmaceutical formulations is limited due to volatility and toxicity [20,21]. It is important to look for extraction solvents that give good yields of bioactive compounds. Aqueous polyethylene glycol (PEG) solvents have recently received a lot of attention from researchers. PEG is water soluble at room temperature and hygroscopic at molecular weights up to 600 [22,23]. PEG has various and wide applications in pharmaceuticals as a solvent, diluent or excipient. Šuran et al. compared the chemical composition of anhydrous PEG400 and ethanolic extracts of poplar-type propolis by maceration. The same compounds were identified in both extracts, and the total amount of phenolic compounds and antioxidant activity were obtained similarly [23]. Thermostability is also one of the important indicators for the application of polyethylene glycol as a solvent for the production of extracts. High temperatures are often used in pharmaceutical technology, for example, for the sterilization of ocular preparations. PEG is used in the manufacture of ocular formulations due to its good biocompatibility [24,25,26].

Topical eye drops are usually characterized by poor absorption of the active substance. Less than 10% of the active substance in topical eye preparations is absorbed by the cornea. This is usually due to the short exposure to the product due to blinking, tear secretion and limited membrane permeability of the cornea [27,28,29]. In order to prolong the effect of ocular formulations on the ocular surface, researchers have devoted considerable time to the development of pharmaceutical ocular formulations with increased viscosity [27,30].

Polymeric materials are capable of forming three-dimensional networks and can retain large amounts of water [27]. Poloxamer is a triblock polymer often used in gel systems, which is composed of poly-oxyethylene/poly-oxy-propylene/poly-oxyethylene blocks [31,32,33]. Poloxamer 407 (P407) is non-toxic, has good compatibility with other chemical compounds and is a suitable solvent for a wide range of active substances [33,34]. P407 molecules tend to aggregate into micelles [33,35]. Although P407 has many positive properties, it forms weak hydrogels. In many cases, other polymeric systems are incorporated into the gel to improve the formulation properties of the system. It is relevant to investigate the wider application of P407 in combination with other polymers. Cellulose derivatives such as hydroxypropyl methyl cellulose (HPMC) or sodium carboxymethyl cellulose (NaCMC) are good gelling agents which, like P407, have a non-irritating, non-toxic effect and can also improve the mucoadhesive properties of the polymeric system. Hyaluronic acid is a linear polysaccharide which has good hydrophilic properties and the ability to form hydrogen bonds with water molecules [36].

Due to its hydrophilic properties, hyaluronic acid (HA) is able to bind large quantities of water. HA also has hydrating properties and a positive effect on wound healing. Due to its beneficial properties, HA is frequently found in ophthalmic preparations, has good biocompatibility and is an endogenous compound found in ocular tissues [36].

The aim of this study is to isolate biologically active compounds from balsam poplar buds using aqueous polyethylene glycol solvents of different concentrations, to formulate extended-release gel systems with the produced balsam poplar bud extract, to evaluate the effect of thermal sterilization on the stability of the active compounds and to determine the effect of the polymer on the quality of eye drops. The study was also aimed at evaluating the stability of the formulated systems, the physicochemical properties of the formulations and the ability to release biologically active compounds.

## 2. Materials and Methods

### 2.1. Materials

Reagents, standards and solvents used were of analytical grade. Purified deionized water prepared with water purification system Milli-Q^®^ (Millipore, Burlington, MA, USA). 96% rectified ethanol (JSC “Vilniaus degtine”, Vilnius, Lithuania). Folin-Ciocalteu’s reagent (Sigma-Aldrich, St. Louis, MO, USA), Acetonitrile (Sigma-Aldrich, Steinheim, Germany), reference standards *p*-coumaric acid, caffeic acid, cinnamic acid (Sigma-Aldrich, Steinheim, Germany), pinobanksin, pinocembrin, galangin, salicin (Sigma-Aldrich, St. Louis, MO, USA). Sodium carbonate (Sigma-Aldrich, Saint-Quentin-Fallavier, France), ABTS (2,20-azino-bis(3-ethylbenzothiazoline-6-sulfonic acid) (Sigma-Aldrich Chemie, Steinheim, Germany). Poloxamer 407 (Sigma-Aldrich, St. Louis, MO, USA), carboxymethyl cellulose sodium salt (Sigma-Aldrich, St. Louis, MO, USA), hydroxypropyl methylcellulose (Sigma-Aldrich, St. Louis, MO, USA), hyaluronic acid (Sigma-Aldrich, St. Louis, MO, USA), polyethylene glycol (Carl Roth, Karlsruhe, Germany).

### 2.2. Plant Material Extraction

Dried balsam poplar buds were purchased from Jadvyga Balvociute’s organic herb farm. Balsam poplar buds were collected in February 2022 in Lithuania. To select a high-quality extract with a good yield of phenolic compounds, the extraction of plant material was carried out using the following solvents: purified water, aqueous 10% PEG400 solution, aqueous 20% PEG400 solution and aqueous 30% PEG400 solution. The ratio of plant raw material and extractant was 1:10. The extraction was carried out in an ultrasonic bath (Bandelin electronic GmbH & Co.KG, Berlin, Germany) at a temperature of 40 °C for 60 min. The obtained extracts are filtered through ashless filter paper and stored in a refrigerator (5 ± 1 °C) until further research.

### 2.3. HPLC Analysis and Antioxidant Activity

The predominant phenolic compounds in poplar buds’ extracts and ophthalmic formulations qualitatively and quantitatively were evaluated by high-performance liquid chromatography (HPLC). Chromatographic system “Waters 2695” with diode array detector “Waters 996”, chromatographic column ACE 5C18, 250 × 4.6 mm was used for analysis. Analysis data was processed by Empower 2 Chromatography Data Software. HPLC eluents consisted of acetonitrile and trifluoroacetic acid. Column temperature was 25 °C, injection volume 10 µL, mobile phase flow rate 1 mL/min, flow time 81 min. The compounds present in the sample were identified by retention time of analytes and reference materials as well as UV absorption (between 250 and 400 nm) [37]. Antioxidant activity was evaluated by HPLC post-column method using ABTS reagent according to Marksa et al. [38]. Test samples were introduced into the HPLC system. ABTS reagent solution (0.5 mL/min) was delivered through a Gilson pump 305 (Middleton, WI, USA). The HPLC-ABTS system used a 3 m reaction loop, with inner diameter 0.25 mm and outer diameter 1.58 mm, at a temperature of 50 °C. During the reaction between the antioxidant and the ABTS reagent, a color change is detected, which was determined using a Waters 247 UV/VIS detector (Waters Corporation, Milford, MA, USA), ACE 5 C18 250 × 4.6 mm column (Advanced Chromatography Technologies, Aberdeen, Scotland) at a wavelength of 650 nm. The results of the post-column reaction antioxidant activity were evaluated according to the Trolox equivalent (254.587–3.977 µg/mL), R^2^ = 0.999. Reference compounds: salicin (R^2^ = 0.9999), *p*-coumaric acid (R^2^ = 0.9999), caffeic acid (R^2^ = 0.9999), chlorogenic acid (R^2^ = 0.9999), cinnamic acid (R^2^ = 0.9999), pinocembrin (R^2^ = 0.9998), pinobanksin (R^2^ = 0.9999), galangin (R^2^ = 0.9999). Results expressed as mean of three measurements and standard deviation (mean ± SD).

### 2.4. Ophthalmic In Situ Gels Formulation

Ophthalmic gels were formulated using: Poloxamer 407 (P407), Hydroxy-propyl-methyl cellulose (HPMC), Sodium carboxymethylcellulose (NaCMC), Hyaluronic acid (HA), purified water, benzalkonium chloride and balsam poplar buds extract. Poloxamer base was prepared by cold method [39]. The appropriate amount of P407 was mixed with the appropriate amount of purified water, the mixture left in the refrigerator for 24 h (5 ± 0.5 °C). HPMC, NaCMC and HA were prepared separately, the appropriate amount of the compound added to the appropriate amount of purified water, the mixtures stirred on a magnetic stirrer at room temperature until homogeneous, and transparent gels were formed. Ophthalmic gels were formed by mixing poloxamer 407 base and HPMC, NaCMC or HA gels with the help of a magnetic stirrer until forming a homogeneous mass. Balsam poplar buds extract and benzalkonium chloride were added to the prepared formulations, and gels mixed to a homogeneous mass with a magnetic stirrer. The formed ophthalmic gels were stored in sealed containers in the dark at room temperature (21 ± 1 °C).

### 2.5. Sterilization of Extracts and Ophthalmic Gels

Sterilization of extracts and ophthalmic gels was performed in a pressure autoclave (System GmbH, Wattenberg, Germany), at 121 °C temperature, 10 min, 15 psi. After sterilization, the formulations were evaluated for appearance, clarity, pH, viscosity and bioactive compounds.

### 2.6. Physicochemical Properties of Ophthalmic Gels

The pH of the formed gels were assessed with a pH meter (766 with Knick SE 104N electrode, Berlin, Germany) at room temperature. The pH meter was calibrated with buffer solutions at pH 4.0–7.0. The viscosity of the formed gels was evaluated with a vibrating viscometer (Vibro viscometer SV-10, A&D Company Ltd., Tokyo, Japan), at room temperature (21 ± 1 °C).

### 2.7. Sol-To-Gel Transition Temperature

The sol-to-gel temperature of experimental ophthalmic formulations was evaluated with a rheometer (Physica MCR 301, Anton Paar GmbH, Graz, Austria) using a standard size concentric cylinder geometry system. Temperature measurement was performed in the range of 20–50 °C, amplitude gamma 0.5%, angular frequency omega 10 rad/s, temperature rate 2 °C per min. The data was processed with Rheoplus software (Anton Paar GmbH, Ostfilder, German). Loss modulus (G″) and storage modulus (G′) were recorded during the temperature change. Sol-to-gel temperature was fixed as the point where storage modulus G′ and loss modulus (G″) cross each other. The storage modulus (G′) describes the ability to store energy elastically and the loss modulus (G″) describes the viscous nature [40].

### 2.8. In Vitro Release Test

The release of active compounds from ophthalmic gel formulations was evaluated using Franz-type diffusion cells and cellulose semipermeable membranes (25 mm, Sigma Aldrich, St. Louis, MO, USA). Cellulose membranes were kept in purified water for 12 h before the test. Purified water was used as an acceptor medium, the volume of the acceptor medium was 10 mL and donor compartment contained 1 g of sample. During the release test, the temperature was maintained at 34 ± 0.5 °C and the medium was constantly stirred with the help of a magnetic stirrer, then 1 mL sample taken from the acceptor medium every hour; the last sample taken after 6 h. The amount of released biologically active compounds was evaluated based on HPLC analysis described earlier.

### 2.9. Antioxidant Activity

Antioxidant activity of test samples by ABTS•+ free radical scavenging method in vitro was performed according to Yim et al.’s described methodology, with certain changes [41]. ABTS stock solution was prepared (0.0548 g ABTS, 0.0095 g K_2_S_2_O_8_ (2 mmol/L), 50 mL purified water). The ABTS stock solution for testing diluted until the absorbance reaches 0.8 ± 0.03 at 734 nm. 3 µL of test samples mixed with 3 mL of ABTS working solution and incubated for 30 min at room temperature. The absorbance of the samples were measured at a wavelength of 734 nm. Results are expressed as a percentage of the inhibition of ABTS solution, ABTS scavenging capacity (%) = (A − B)/A × 100, when A—absorbance of ABTS solution, B—absorbance of ABTS solution and sample.

### 2.10. Statistical Analysis

The results of the tests presented as the mean and standard deviation of three measurements (mean ± SD). The statistical significance of the comparative results were evaluated by one-way ANOVA, Tukey’s test. Results considered statistically significant when *p* < 0.05. Data were processed and graphically presented using SigmaPlot 13.0 (Systat Software, San Jose, CA, USA) and IBM SPSS Statistics 27 (SPSS Inc., Chicago, IL, USA).

## 3. Results

### 3.1. HPLC Analysis of the Extracts

The highest number of active compounds was obtained in the 30%PEG aqueous extract of balsam poplar buds (1209.31 µg/mL). When using aqueous PEG400 mixtures, a statistically significantly higher (*p* < 0.05) number of active compounds was released than in the aqueous extract (HOH) (Table 1A). The highest amount of salicin was extracted from the extracts using an aqueous 30% PEG400 solvent (138.00 ± 9.61 µg/mL). From our identified compounds, *p*-coumaric acid is predominant in all extracts. Statistically significantly lower (*p* < 0.05) amounts of caffeic and cinnamic acids were detected compared to *p*-coumaric acid. The number of phenolic acids in aqueous extracts of PEG400 is statistically significantly higher (*p* < 0.05) than in aqueous extracts (HOH). In order to apply the extracts for the production of ophthalmic topical preparations, the extracts were sterilized by autoclaving and the chemical composition after sterilization was evaluated. The results of the study are presented in Table 1B. According to the results obtained, it was observed, that the chemical composition of the extracts did not change statistically significantly (*p* > 0.05) after heat sterilization. In the light of the results obtained, an aqueous 30% PEG400 extract was selected for the production of ophthalmic topical formulations, which has a good yield of active compounds.

### 3.2. Composition of Formulations Ant Their Antioxidant Activity

**Figure 1:**
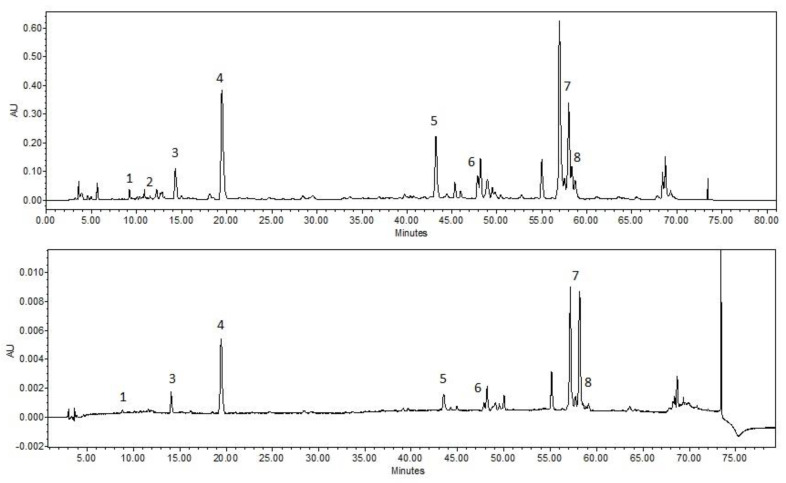
HPLC analysis of the chemical composition of of 30% aqueous PEG400 balsam poplar buds extract and BC12 gel with balsam poplar buds extract. Above—chromatogram of 30% aqueous PEG400 balsam poplar buds extract after sterilization, Below—chromatogram of BC12 gel after sterilization. 1. salicin, 2. chlorogenic acid, 3. caffeic acid, 4. *P*-coumaric acid, 5. cinnamic acid, 6. pinobanksin, 7. pinocembrin, 8. galangin. The chromatogram is presented at a wavelength of 290 nm.

Formulations were produced with poloxamer 407 (8–12%) as the main gelling agent. Additional gelling agent HPMC was used in formulations BH8-BH15, NaCMC in formulations BC8-BC15 and HA in formulations BHA8-BHA15. All formulations use benzalkonium chloride as a preservative. All formulations incorporate 5% balsam poplar buds extract, which is produced using an aqueous 30% PEG400 solvent (Table 2). All topical ophthalmic formulations produced identified the same active compounds as the incorporated balsam poplar bud extract (Figure 1).

**Figure 2:**
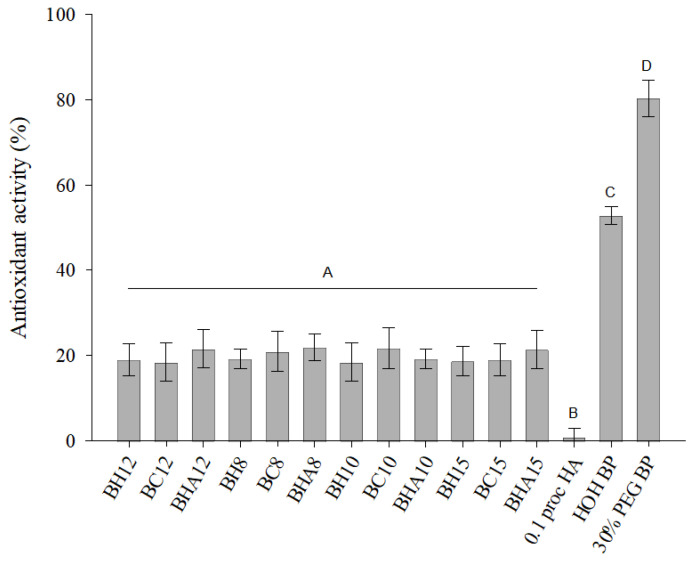
Antioxidant activity of prepared formulations, 0.1% Hyaluronic acid solution, aqueous and aqueous 30% PEG400 extracts of balsamic poplar buds by ABTS method. Different letters indicate statistically significant differences between the antioxidant activity of the samples (*p* < 0.05).

From the results presented in Figure 2, it can be seen that the prepared formulations with balsam poplar buds extract showed antioxidant activity by the ABTS method. Formulations produced without the inserted poplar buds extract did not show antioxidant activity. Antioxidant activity of hyaluronic acid 0.1% solution was not detected. No statistically significant difference (*p* > 0.05) was found between the antioxidant activity of the produced formulations. The antioxidant activity of the formulations by the ABTS method prevailed from 18.51 ± 4.53% to 21.94 ± 3.20%. The 30% PEG400 balsamic poplar buds extract showed statistically significantly stronger (*p* < 0.05) antioxidant activity (80.35 ± 4.37%) compared to the aqueous balsam poplar buds extract (52.82 ± 2.21%).

**Figure 3:**
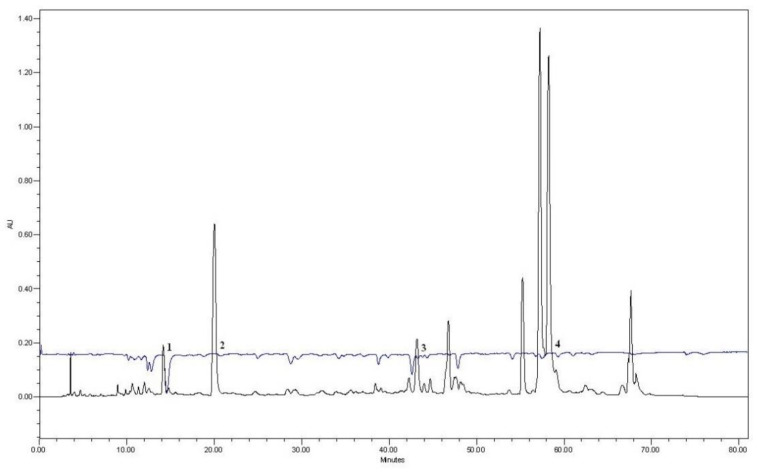
HPLC chromatogram of 30% aqueous PEG400 balsam poplar buds extract combined with their respective post-column ABTS chromatogram which presented as negative peaks (1—caffeic acid, 2—*p*-coumaric acid, 3—cinnamic acid, 4—galangin).

The antioxidant activity of an aqueous 30% PEG400 balsam poplar buds extract was evaluated by the post-column method using the ABTS reagent. It can be seen in the results that caffeic acid, *p*-coumaric acid, cinnamic acid and galangin showed antioxidant activity in the extract (Figure 3).

### 3.3. Physicochemical Properties

**Figure 4:**
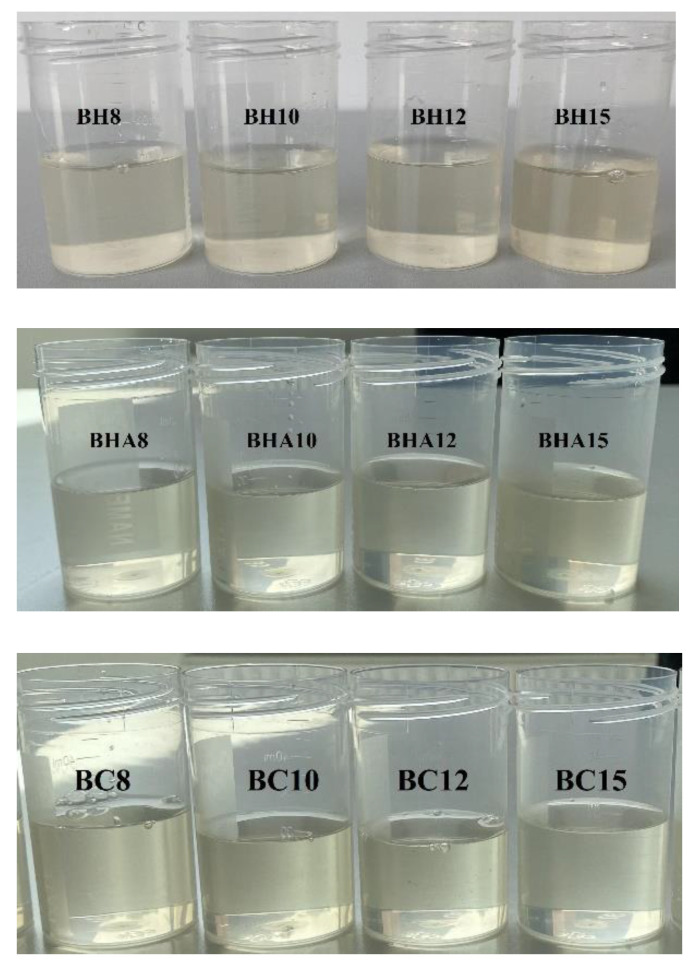
Ophthalmic formulations with balsam poplar buds extract after sterilization.

All the ophthalmic formulations produced present liquid consistency at room temperature. All the produced formulations were transparent, without visually visible mechanical impurities, with a typical light yellowish color (Figure 4).

In order to evaluate the stability of ophthalmic formulations for sterilization, the physicochemical properties of the formulations before sterilization and after sterilization were evaluated (Table 3). After evaluating the viscosity, pH and appearance of the formulations, it can be stated that there is no statistically significant (*p* > 0.05) change in the physicochemical parameters when comparing the ophthalmic formulations before sterilization and after autoclave sterilization.

Based on the results of the physicochemical properties, the eye drop formulations were divided into three groups: I—In situ gels (BH12, BC12, BHA12), II—Gelled eye drops (BH8, BH10, BC8, BC10, BHA8, BHA10), III—Solutions with a viscosity of more than 40 mPa·s (BH15, BC15, BHA15) (Table 3). The pH values of the formulations before sterilization ranged from 6.46 ± 0.06 to 6.70 ± 0.04, after sterilization ranged from 6.42 ± 0.04 to 6.62 ± 0.05. According to the results of the tests, an increase in the viscosity of the formulations was observed with increasing concentration of P407 in the formulation. The highest viscosity was observed for the formulations (BH15, BC15, BHA15) containing 15% poloxamer (from 62.27 ± 6.45 mPa·s to 67.27 ± 5.99 mPa·s—before sterilization, from 55.27 ± 4.72 mPa·s to 63.80 ± 6.66 mPa·s—after sterilization). The rheological test and the evaluation of the gelation point of the formulations after sterilization showed that formulations BH12, BC12 and BHA12 exhibited in situ gel properties as their gelation temperature was in the range of 34.30 ± 1.35 °C to 37.90 ± 1.11 °C. Formulations BH15, BC15 and BHA15 showed lower gelation temperatures compared to in situ gels, ranging from 27.10 ± 1.10 °C to 28.70 ± 1.28 °C. Formulations using 8–10% poloxamer had temperatures above 50 °C.

### 3.4. Phenolic Acids Release

**Figure 5:**
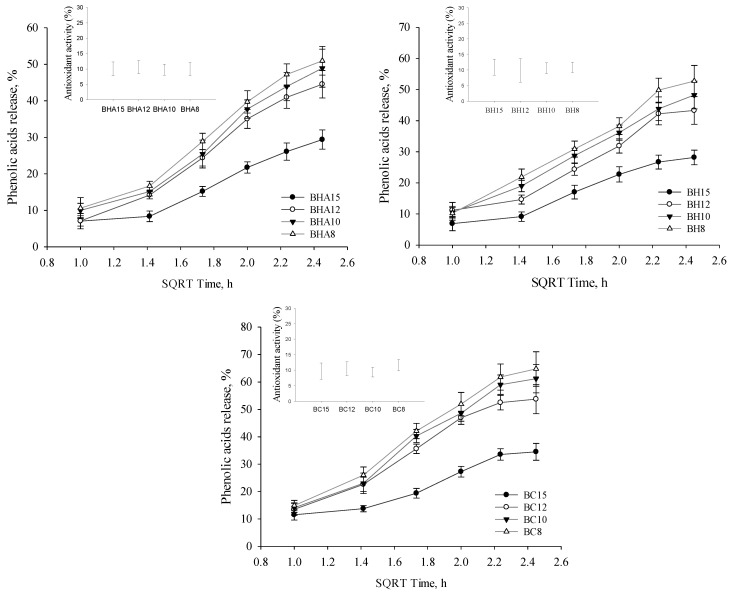
Percentage release of phenolic acids (sum of *p*-coumaric, cinnamic and caffeic acids) versus square root of time from all tested ophthalmic formulations.Subfigure shows antioxidant activity by ABTS method from the fractions collected during the release test after 6 h (mean ± SD, *n* = 3).

After performing the in vitro release of the formed and sterilized ophthalmic gel preparations, it was found that formulations based on 15% P407 release a statistically significantly lower (*p* < 0.05) amount (from 28.18% to 34.51%) of phenolic acids (sum of *p*-coumaric acid, cinnamic acid, caffeic acid) compared to formulations based on 8–12% P407 (Figure 5). Among the formulations based on 8–10% P407, the amount of released phenolic acids was not statistically significantly different (*p* > 0.05). The highest number of phenolic acids was released by gelled eye drops BC8 (64.75%), BC10 (61.21%) and in situ gel BC12 (53.78%). During the study, the antioxidant activity of the fractions obtained during the release of the formulations (after 6 h) was evaluated (Figure 5). The fractions showed weak antioxidant activity (From 9.37% to 11.61%).

### 3.5. Salicin Release

**Figure 6:**
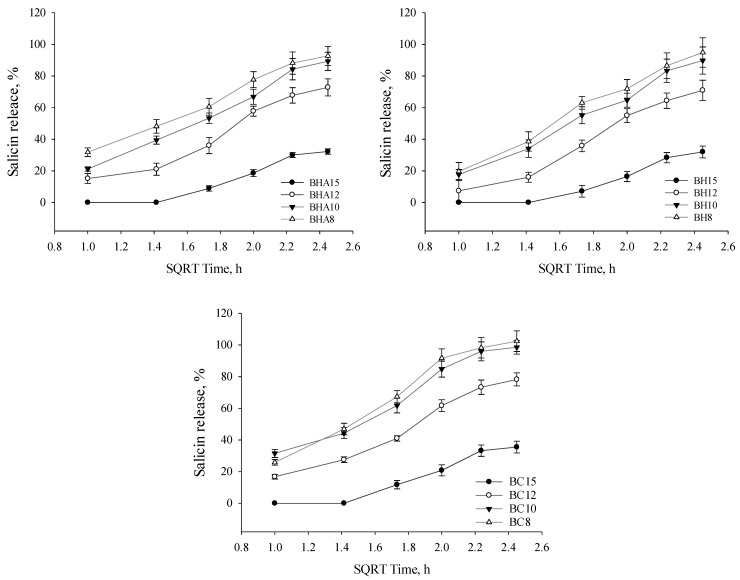
Percentage release of salicin verses square root of time from all tested ophthalmic formulations (mean ± SD, *n* = 3).

The released amount of salicin in the manufactured extended-release eye drops was evaluated (Figure 6). The highest salicin content was obtained from gelled eye drops BC8 (102.43%), BC10 (98.56%) and BHA8 (92.76%). A statistically significantly lower (*p* < 0.05) amount of salicin was released from high viscosity solutions BH15, BC15 and BHA15 compared to lower viscosity formulations. No statistically significant (*p* > 0.05) amount of released salicin was determined in formulations based on 8% and 10% P407.

### 3.6. Oftalmic Gels Stability

The stability of sterilized ophthalmic formulations after 30 days was evaluated. The formulations were stored at room temperature (21 ± 1 °C). Formulations, viscosity, pH, number of active compounds and appearance were evaluated. According to the obtained results, which are presented in Table 4, it is observed that the pH and viscosity of the formulations did not change significantly. The formulations remained clear, slightly yellowish in color, with no visible sediment.

## 4. Discussion

### 4.1. Extraction of Phenolic Compounds from Balsam Poplar Buds

In the selection of solvents for the extraction of balsam poplar buds, aqueous solvents were chosen, which are acceptable for use in ophthalmic formulations. In this study, the quality of the extracts was evaluated by determining the phenolic acids, flavonoids and salicin content. Salicin is one of active ingredients of the broad Salicaceae woody plant family [42]. This compound has anti-inflammatory properties [43,44]. Salicin was the original source of aspirin. Aspirin, also known as acetylsalicylic acid (ASA), is a medication used to reduce pain, fever or inflammation [45,46,47]. The mechanism of action is thought to be through the combined anti-inflammatory effects of salicin and flavonoids [48]. Salicylates are also used in topical ophthalmic preparations [43,49,50]. Aspirin eye drops were studied, having anti-cataractic activity in patients with galactosemic cataract [51]. Eye drops with salicylic acid had an effective anti-inflammatory effect in glaucoma patients. They reduced inflammation, improved tear film quality and quantity, and did not cause a significant increase in intraocular pressure [52]. The study results showed that the selected aqueous PEG solutions and purified water are suitable solvents for the isolation of salicin from plant material. The results of previous studies have shown that purified water extracts a higher amount of salicin from poplar buds compared to ethanol solvents [14,53]. The results of the present study showed that the addition of PEG does not adversely affect the yield of salicin. Flavonoids were identified in the extracts, of which pinobanksin was found in the highest amounts and galangin and pinocembrin in lower amounts. Researchers attribute the antimicrobial activity to these identified flavonoids in the propolis raw material [54,55,56]. Poplar buds often are the precursor plant of propolis, and therefore most of the active compounds correlate between the extracts of these raw materials. Like propolis extracts, poplar bud extracts are dominated by *p*-coumaric acid [14,37]. The highest levels of this phenolic acid are also found in our extracts.

### 4.2. Antioxidant Activity of Ophthalmic Formulations

Research results show that eye drops with *p*-coumaric acid protect eye tissues, thereby reducing the harmful effects of UVB radiation due to their ability to bind free radicals and their antioxidant properties [57,58]. UV-induced oxidation damage seems to play a major role in a number of specific pathological conditions of intraocular tissues, such as cataract formation and retinal degeneration [59,60]. The results showed that, among the identified active compounds in poplar bud extracts, caffeic acid, *p*-coumaric acid, cinnamic acid and galangin showed the highest antioxidant activity by post-column ABTS HPLC analysis. The results showed that the eye drop formulations produced showed weak antioxidant activity, which can be attributed to the low extract concentration in the formulations. The balsam poplar bud extract produced showed antioxidant activity by ABTS in vitro. The results of the antioxidant activity test confirm the importance of choosing the solvent for the extraction of the active compounds. It was found that the aqueous 30% PEG400 poplar bud extract had a stronger antioxidant activity compared to the aqueous extract. Scientists have found, that hyaluronic acid derivatives have antioxidant activity [61]. In a study conducted by Chunlin et al., it was found that low molecular weight hyaluronic acid derivatives exhibited antioxidant activity and that the compounds were able to reduce lipid peroxidation [62]. In this experiment, we investigated the antioxidant activity of 0.1% (~1.5–1.8 × 10^6^ Da) hyaluronic acid in the formulations. The results did not show any antioxidant activity of the 0.1% hyaluronic acid solution using the ABTS method in vitro. Hyaluronic acid was chosen as a gelling agent with good biocompatibility in the simulated eye drop formulations [36] Hyaluronic acid is also known to have a moisturizing effect [63,64].

### 4.3. Sterilization of Formulations

Lysozyme in eye tears is known to have antimicrobial properties, killing Gram-positive bacteria under non-pathological conditions [65]. In the case of eye diseases, the amount of lysozyme in the tear fluid is reduced, making the eye vulnerable to infection by micro-organisms. The use of non-sterile eye drops can lead to serious consequences, in some cases even to loss of vision, which is why one of the important requirements for eye drops is sterility. For ophthalmic preparations, the choice of the sterilization method is one of the most important technological steps [66]. Various sterilization methods are available, such as treatment with chemical compounds (ethylene oxide), sterile filtration, irradiation or autoclaving [67]. One of the most frequently used sterilization methods is sterilization by moist heat-autoclaving. In this study, all formulations were sterilized using this method. In the case of autoclaving, it is important to assess the stability of the eye drop components at high temperatures. In the present study, the active compounds were evaluated before and after sterilization in poplar bud extracts and ocular formulations. The available results showed that the chemical composition of the sterilized extracts did not change statistically significantly (*p* > 0.05). This indicates that the active compounds in the extracts are thermostable and therefore autoclaving is one of the possible sterilization methods for the production of balsam poplar bud extracts. In addition, after sterilization, the ocular formulations with different compositions remained transparent without significant changes in viscosity, pH, and active compound content. There is evidence in the scientific literature that polymers such as P407, NaCMC and HPMC can be autoclaved [68,69,70]. Autoclaving hyaluronic acid at high temperatures often results in lower molecular weight derivatives, which may affect the viscosity of the formulation [71]. HA is an unstable molecule at temperatures above 100 °C due to damage to the interchain bonds [72]. Higher molecular weight HA derivatives will not necessarily suffer a pronounced negative effect because of sterilization. According to the HA hydrogel patent, for HA with a molecular weight of 1 to 3 MDa, steam sterilization at 126 °C for 15 min to 30 min is a possible method of sterilization, but the high temperature affects the degradation of HA [73]. Considering the potential degradation of hyaluronic acid at high temperatures, autoclaving for 10 min at 121 °C was chosen. In our study, the selected sterilization conditions ensured the stability of the formulations as their physicochemical properties and the content of active compounds did not change statistically significantly (*p* > 0.05).

### 4.4. Physicochemical Properties of the Formulations

Poloxamer gels can be used in a variety of biopharmaceutical applications [74]. Recently, much attention has been paid to thermosetting in situ gels, which are designed for intraocular drug delivery and can prolong the duration of action [30]. P407 gels are transparent, which is an important feature of ocular formulations that does not interfere with vision [27]. In order to improve the formulation properties of the P407 hydrogel, it has been chosen to add more polymers to the gels. Cellulose polymer compounds are gaining more and more scientific attention [75] and we have chosen to combine HPMC and NACMC polymers with P407. The rheological properties of the polymer systems formed are receiving increasing attention. Out of 12 formulations produced, three formulations matched the in situ gel characteristics. In the study, the choice of P407 concentrations of 8, 10, 12 and 15% in the formulations resulted in an increase in viscosity with increasing polymer content. The formulations were divided into three groups according to the viscosity results: in situ gels; gelled eye drops; solutions with a viscosity greater than 30 mPa·s. The eye can tolerate viscosities of 5 to 1000 mPa·s before gelation and about 50 to 50,000 mPa·s after gelation [76]. The minimum shear viscosity of eye drops required to maintain precorneal residence in man has been reported to be 10 mPa·s [77]. Considering the viscosity of the formulated ocular preparations, the lowest viscosity was observed for the formulations of group II (15.60 ± 2.26 mPa·s–23.23 ± 4.13 mPa·s) (Table 3B). Higher viscosities were characteristic of groups’ I and III formulations. Group I formulations had a viscosity from 31.20 ± 3.38 mPa·s to 32.03 ± 4.35 mPa·s and exhibited a required gelation temperature of 34.3 to 37.9 °C characteristic of in situ gels (Table 3B). According to the scientific literature, the temperature at the surface of the eye can range approximately from 33 to 36 °C [78,79,80,81]. The correct in situ gelation temperature is important for the ease of formulation, as the gel is low viscosity at room temperature and will only gel once it reaches the ocular surface. Other formulations had either too low (from 27.1 ± 1.10 °C to 28.7 ± 1.28 °C) or too high (over 50 °C) gelation temperature. Group III gels were formulations with a higher viscosity than in situ gels and a low gelation temperature. All formulations produced had a viscosity tolerable to the ocular surface [76]. The pH of the formulations produced ranged from 6.42 ± 0.04 to 6.62 ± 0.05. All formulations had a suitable pH value [82] and it can be concluded that the extract does not adversely affect the pH value.

### 4.5. Release of the Active Compounds

Polymeric systems allow prolonged action of the drug on the ocular surface [83,84]. The aim of this experimental study was to investigate the kinetics of the release of active compounds from the formulations produced in vitro. The higher viscosity of the formulations prolongs the residence time of the formulation on the ocular surface, which allows prolongation of the drug action [85]. The results of our study showed that phenolic acids (caffeic acid, *p*-coumaric acid and cinnamic acid) and salicin were released in the lowest amount in the group III formulations after 6 h of testing, and that these formulations had the highest viscosity when compared to the groups I and II formulations. The released phenolic acid content was statistically significantly lower in group III formulations compared to groups I and II formulations. The highest amounts of phenolic acids and salicin were released from group II formulations which had the lowest viscosity. When the formulations were based on two polymer combinations, P407/HPMC, P407/HA and P407/NaCMC, all formulations showed similar release results for the active compounds, respectively. It can be concluded that the quality of release is influenced by the concentration of P407. Yidan Wei et al. investigated the effect of the composition of P407-based in situ type gels on the release of betaxolol hydrochloride. The concentration of P407 determined the release of betaxolol hydrochloride, whereas the addition of HPMC to the P407 base resulted in a further dependence of the drug release on the concentration of P407 [86]. As the concentration of P407 in the formulations decreased from 15% to 8%, the percentage release of phenolic acids increased. Increasing poloxamer concentration increases the density of micelle aggregation, resulting in a more rigid gel structure, which reduces the release of the active substance [27,87]. It is important to consider that higher viscosity and in situ formulations may prolong the residence time of the formulation on the ocular surface [88]. Low viscosity eye drops are easier to remove from the surface of the eye by flowing and mechanical blinking [89].

### 4.6. Stability of Formulations

Stability of ophthalmic formulations is a valid aspect and it is important that the physicochemical characteristics of the formulation remain significantly unchanged for the appropriate period of storage or use from the start of manufacture [67]. The stability of ophthalmic formulations depends on a variety of factors, such as the stability of the active substance, temperature, storage conditions or excipients [90]. The formulations were stored at room temperature for 30 days after sterilization. After 30 days, the pH and viscosity of the formulations did not change statistically significantly (*p* > 0.05). The ocular formulations remained light yellow in color, transparent and the total content of active compounds did not change statistically significantly (*p* > 0.05).

## 5. Conclusions

Prolonged-action eye formulations are made on the basis of P407/HPMC, P407/NaCMC and P407/HA with the addition of aqueous 30% PEG400 balsam poplar buds extract. Extracts and formulations remained stable after autoclaving. Formulations based on 12% P407 were characterized by the properties of in situ gels, with a gelation temperature close to the temperature of the eye. As the concentration of polymer P407 in the formulations increases, the viscosity of the formulations increases. The released number of active compounds depends on the concentration of polymer P407 in the eye formulation. The sterilized ophthalmic formulations were stable for 30 days.

## Figures and Tables

**Table 1:** HPLC analysis of balsam poplar buds aqueous, aqueous 10% PEG400, aqueous 20% PEG400 and aqueous 30% PEG400 extracts. A—before sterilization, B—after sterilization. The data presented as mean (µg/mL) and standard deviation (SD), *n* = 3.

**A**	**HOH**		**PEG400 10%**		**PEG400 20%**		**PEG400 30%**	
	**µg/mL**	**SD**	**µg/mL**	**SD**	**µg/mL**	**SD**	**µg/mL**	**SD**
Salicin	135.80	12.35	108.91	9.91	117.95	10.73	138.00	9.61
Chlorogenic acid	1.41	0.10	1.39	0.10	1.84	0.13	2.95	0.22
Caffeic acid	38.68	2.75	91.90	6.54	90.17	6.42	121.55	9.01
*P*-coumaric acid	189.05	13.60	441.13	31.73	460.64	33.13	655.69	50.02
Cinnamic acid	19.22	1.42	30.74	2.27	44.10	3.26	85.11	5.49
Pinobanksin	2.92	0.29	61.67	6.23	91.64	9.25	174.43	10.72
Pinocembrin	0.00	0.00	2.15	0.20	6.05	0.55	12.97	1.15
Galangin	1.16	0.12	18.32	1.30	17.22	1.57	18.61	1.38
Total amount of active compounds	388.24		756.21		829.61		1209.31	
**B**	**HOH**		**PEG400 10%**		**PEG400 20%**		**PEG400 30%**	
	**µg/mL**	**SD**	**µg/mL**	**SD**	**µg/mL**	**SD**	**µg/mL**	**SD**
Salicin	124.86	11.36	101.52	8.26	129.05	11.74	144.83	13.18
Chlorogenic acid	1.50	0.10	1.29	0.10	2.03	0.14	3.02	0.34
Caffeic acid	44.13	3.14	85.01	5.92	101.02	7.19	133.39	9.50
*P*-coumaric acid	201.92	14.52	404.30	33.45	491.55	35.36	681.98	49.05
Cinnamic acid	15.92	1.18	27.27	1.57	40.46	2.99	81.41	6.02
Pinobanksin	3.09	0.31	55.17	5.46	83.07	8.39	157.21	15.88
Pinocembrin	0.00	0.00	2.11	0.17	4.16	0.38	10.23	0.93
Galangin	1.05	0.10	16.30	1.40	18.74	1.69	19.13	2.31
Total amount of active compounds	392.47		692.96		870.08		1231.20	

**Table 2:** Compositions of ophthalmic formulations with balsam poplar buds 30% aqueous PEG400 extract.

Formulation (%)	P407	CMC	HPMC	HA	Balsam Poplar Buds Extract	Benzalkonium Chloride	Purified Water
BH8	8	-	0.5	-	5	0.002	Ad 100
BH10	10	-	0.5	-	5	0.002	Ad 100
BH12	12	-	0.5	-	5	0.002	Ad 100
BH15	15	-	0.5	-	5	0.002	Ad 100
BC8	8	0.5	-	-	5	0.002	Ad 100
BC10	10	0.5	-	-	5	0.002	Ad 100
BC12	12	0.5	-	-	5	0.002	Ad 100
BC15	15	0.5	-	-	5	0.002	Ad 100
BHA8	8	-	-	0.1	5	0.002	Ad 100
BHA10	10	-	-	0.1	5	0.002	Ad 100
BHA12	12	-	-	0.1	5	0.002	Ad 100
BHA15	15	-	-	0.1	5	0.002	Ad 100

**Table 3:** Physicochemical properties of ophthalmic gels’ formulations with balsam poplar buds extract before (A) and after (B) sterilization (mean, SD, *n* = 3).

**A**	**pH**	**SD**	**Vsc, mPa·s 22 ± 1 °C**		**SD**		**Appearance**
BH12	6.60	0.06	33.90		4.36		Clear/yellowish
BC12	6.64	0.07	32.57		4.23		Clear/yellowish
BHA12	6.64	0.09	36.03		3.45		Clear/yellowish
BH8	6.47	0.04	15.57		2.85		Clear/yellowish
BC8	6.46	0.06	17.27		2.45		Clear/yellowish
BHA8	6.55	0.04	18.47		2.61		Clear/yellowish
BH10	6.50	0.04	18.03		2.18		Clear/yellowish
BC10	6.46	0.07	24.27		2.70		Clear/yellowish
BHA10	6.50	0.06	23.27		2.90		Clear/yellowish
BH15	6.70	0.04	67.27		5.99		Clear/yellowish
BC15	6.62	0.05	66.80		3.99		Clear/yellowish
BHA15	6.67	0.06	62.27		6.45		Clear/yellowish
**B**	**pH**	**SD**	**Vsc, mPa·s 21 ± 1 °C**	**SD**	**Sol-to-gel**	**SD**	**Appearance**
BH12	6.49	0.06	32.03	4.35	36.4	0.9	Clear/yellowish
BC12	6.50	0.07	31.67	5.20	34.3	1.35	Clear/yellowish
BHA12	6.56	0.04	31.20	3.38	37.9	1.11	Clear/yellowish
BH8	6.40	0.06	16.23	2.29	>50 °C	-	Clear/yellowish
BC8	6.40	0.07	15.60	2.26	>50 °C	-	Clear/yellowish
BHA8	6.58	0.01	17.37	2.67	>50 °C	-	Clear/yellowish
BH10	6.42	0.04	23.23	4.13	>50 °C	-	Clear/yellowish
BC10	6.44	0.04	21.77	2.29	>50 °C	-	Clear/yellowish
BHA10	6.49	0.06	22.20	5.13	>50 °C	-	Clear/yellowish
BH15	6.62	0.05	63.80	6.66	27.4	1.1	Clear/yellowish
BC15	6.56	0.04	61.67	6.27	28.7	1.28	Clear/yellowish
BHA15	6.61	0.02	55.27	4.72	27.1	1.1	Clear/yellowish

**Table 4:** Physicochemical properties of ophthalmic gels formulations after 30 days (mean, SD, *n* = 3).

	pH	SD	Vsc, mPa·s 21 ± 1 °C	SD	Active Compounds %	SD	Appearance
BH12	6.54	0.08	30.93	3.15	98.95	4.07	Clear/yellowish
BC12	6.57	0.06	29.42	2.47	98.75	2.94	Clear/yellowish
BHA12	6.53	0.07	29.71	5.1	97.68	4.88	Clear/yellowish
BH8	6.5	0.07	13.36	2.93	98.36	6.81	Clear/yellowish
BC8	6.5	0.04	12.96	1.46	99.2	2.21	Clear/yellowish
BHA8	6.49	0.08	14.4	3.96	98.81	3.46	Clear/yellowish
BH10	6.48	0.04	18.17	3.68	97.19	4.17	Clear/yellowish
BC10	6.49	0.08	17.5	2.48	96.94	4.87	Clear/yellowish
BHA10	6.48	0.09	18.31	4.49	98.34	2.88	Clear/yellowish
BH15	6.6	0.08	61.33	6.14	97.53	5.69	Clear/yellowish
BC15	6.67	0.07	60.52	4.34	99.13	4.15	Clear/yellowish
BHA15	6.68	0.07	54.38	2.76	98.56	3.75	Clear/yellowish

## Data Availability

All data are available in a publicly accessible repository.

## References

[1] Yapar E.A., Durgun M., Esentürk I., Güngör S., Özsoy Y. (2022). Herbal bioactives for ocular drug delivery systems. Herbal Bioactive-Based Drug Delivery Systems.

[2] Krstić L., González-García M.J., Diebold Y. (2021). Ocular Delivery of Polyphenols: Meeting the Unmet Needs. Molecules.

[3] Choi W., Lee J.B., Cui L., Li Y., Li Z., Choi J.S., Lee H.S., Yoon K.C. (2016). Therapeutic Efficacy of Topically Applied Antioxidant Medicinal Plant Extracts in a Mouse Model of Experimental Dry Eye. Oxidative Med. Cell. Longev..

[4] Deng R., Hua X., Li J., Chi W., Zhang Z., Lu F., Zhang L., Pflugfelder S.C., Li D.-Q. (2015). Oxidative Stress Markers Induced by Hyperosmolarity in Primary Human Corneal Epithelial Cells. PLoS ONE.

[5] Pokkalath A.S., Sawant A., Sawarkar S.P. (2021). Herbal medicine for ocular diseases: An age old therapy and its future perspective. J. Drug Deliv. Sci. Technol..

[6] Yang C.-C., Su S.-H., Ho T.-J. (2021). Retrospective evaluation of the curative effect of traditional Chinese medicine on dry eye disease. Tzu Chi Med. J..

[7] Seen S., Tong L. (2018). Dry eye disease and oxidative stress. Acta Ophthalmol..

[8] Bigagli E., Cinci L., D’Ambrosio M., Luceri C. (2017). Pharmacological activities of an eye drop containing Matricaria chamomilla and Euphrasia officinalis extracts in UVB-induced oxidative stress and inflammation of human corneal cells. J. Photochem. Photobiol. B Biol..

[9] Arana L., Salado C., Vega S., Aizpurua-Olaizola O., de la Arada I., Suarez T., Usobiaga A., Arrondo J.L.R., Alonso A., Goñi F.M. (2015). Solid lipid nanoparticles for delivery of Calendula officinalis extract. Colloids Surf. B Biointerfaces.

[10] Fraunfelder F.W. (2004). Ocular side effects from herbal medicines and nutritional supplements. Am. J. Ophthalmol..

[11] Anjum S.I., Ullah A., Khan K.A., Attaullah M., Khan H., Ali H., Bashir M.A., Tahir M., Ansari M.J., Ghramh H.A. (2019). Composition and functional properties of propolis (bee glue): A review. Saudi J. Biol. Sci..

[12] Zhang C.-P., Zheng H.-Q., Liu G., Hu F.-L. (2011). Development and validation of HPLC method for determination of salicin in poplar buds: Application for screening of counterfeit propolis. Food Chem..

[13] Dezmirean D.S., Paşca C., Moise A.R., Bobiş O. (2020). Plant Sources Responsible for the Chemical Composition and Main Bioactive Properties of Poplar-Type Propolis. Plants.

[14] Stanciauskaite M., Marksa M., Liaudanskas M., Ivanauskas L., Ivaskiene M., Ramanauskiene K. (2021). Extracts of Poplar Buds (*Populus balsamifera* L., *Populus nigra* L.) and Lithuanian Propolis: Comparison of Their Composition and Biological Activities. Plants.

[15] Zielińska A., Soles B.B., Lopes A.R., Vaz B.F., Rodrigues C.M., Alves T.F.R., Klensporf-Pawlik D., Durazzo A., Lucarini M., Severino P. (2020). Nanopharmaceuticals for Eye Administration: Sterilization, Depyrogenation and Clinical Applications. Biology.

[16] Garibaldi B.T., Reimers M., Ernst N., Bova G., Nowakowski E., Bukowski J., Ellis B.C., Smith C., Sauer L., Dionne K. (2017). Validation of Autoclave Protocols for Successful Decontamination of Category A Medical Waste Generated from Care of Patients with Serious Communicable Diseases. J. Clin. Microbiol..

[17] Cooper R.C., Yang H. (2019). Hydrogel-based ocular drug delivery systems: Emerging fabrication strategies, applications, and bench-to-bedside manufacturing considerations. J. Control. Release.

[18] Galante R., Pinto T.D.J.A., Colaco R., Serro A.P. (2018). Sterilization of hydrogels for biomedical applications: A review. J. Biomed. Mater. Res. Part B Appl. Biomater..

[19] Cvjetko Bubalo M., Vidović S., Radojčić Redovniković I., Jokić S. (2018). New perspective in extraction of plant biologically active compounds by green solvents. Food Bioprod. Process..

[20] Castillo F., Hernández D., Gallegos G., Mendez M., Rodríguez R., Reyes A., Aguilar C.N. (2010). In vitro antifungal activity of plant extracts obtained with alternative organic solvents against Rhizoctonia solani Kühn. Ind. Crop. Prod..

[21] Płotka-Wasylka J., Rutkowska M., Owczarek K., Tobiszewski M., Namieśnik J. (2017). Extraction with environmentally friendly solvents. TrAC Trends Anal. Chem..

[22] Chen J., Spear S.K., Huddleston J.G., Rogers R.D. (2005). Polyethylene glycol and solutions of polyethylene glycol as green reaction media. Green Chem..

[23] Šuran J., Cepanec I., Mašek T., Starčević K., Gajger I.T., Vranješ M., Radić B., Radić S., Kosalec I., Vlainić J. (2021). Nonaqueous Polyethylene Glycol as a Safer Alternative to Ethanolic Propolis Extracts with Comparable Antioxidant and Antimicrobial Activity. Antioxidants.

[24] Hoffmann M.M. (2022). Polyethylene glycol as a green chemical solvent. Curr. Opin. Colloid Interface Sci..

[25] Llorens E., Ibañez H., del Valle L., Puiggalí J. (2015). Biocompatibility and drug release behavior of scaffolds prepared by coaxial electrospinning of poly(butylene succinate) and polyethylene glycol. Mater. Sci. Eng. C.

[26] Vrandečić N.S., Erceg M., Jakić M., Klarić I. (2010). Kinetic analysis of thermal degradation of poly(ethylene glycol) and poly(ethylene oxide)s of different molecular weight. Thermochim. Acta.

[27] Soliman K.A., Ullah K., Shah A., Jones D.S., Singh T.R. (2019). Poloxamer-based in situ gelling thermoresponsive systems for ocular drug delivery applications. Drug Discov. Today.

[28] Tundisi L., Mostaço G., Carricondo P., Petri D. (2021). Hydroxypropyl methylcellulose: Physicochemical properties and ocular drug delivery formulations. Eur. J. Pharm. Sci..

[29] Kim H., Woo S. (2021). Ocular Drug Delivery to the Retina: Current Innovations and Future Perspectives. Pharmaceutics.

[30] Majeed A., Khan N.A. (2019). Ocular in situ gel: An overview. J. Drug Deliv. Ther..

[31] Ricci E.J., Lunardi L.O., Nanclares D.M.A., Marchetti J.M. (2005). Sustained release of lidocaine from Poloxamer 407 gels. Int. J. Pharm..

[32] Dumortier G., Grossiord J.L., Agnely F., Chaumeil J.C. (2006). A Review of Poloxamer 407 Pharmaceutical and Pharmacological Characteristics. Pharm. Res..

[33] da Silva J.B., Cook M.T., Bruschi M.L. (2020). Thermoresponsive systems composed of poloxamer 407 and HPMC or NaCMC: Mechanical, rheological and sol-gel transition analysis. Carbohydr. Polym..

[34] Ban E., Park M., Jeong S., Kwon T., Kim E.-H., Jung K., Kim A. (2017). Poloxamer-Based Thermoreversible Gel for Topical Delivery of Emodin: Influence of P407 and P188 on Solubility of Emodin and Its Application in Cellular Activity Screening. Molecules.

[35] Jiang Q., Zhang P., Li J. (2020). Elucidation of Colloid Performances of Thermosensitive In Situ–Forming Ophthalmic Gel Formed by Poloxamer 407 for Loading Drugs. J. Pharm. Sci..

[36] Zhang X., Wei D., Xu Y., Zhu Q. (2021). Hyaluronic acid in ocular drug delivery. Carbohydr. Polym..

[37] Ramanauskienė K., Savickas A., Inkėnienė A., Vitkevičius K., Kasparavičienė G., Briedis V., Amšiejus A. (2009). Analysis of content of phenolic acids in Lithuanian propolis using high-performance liquid chromatography technique. Medicina.

[38] Marksa M., Radušienė J., Jakštas V., Ivanauskas L., Marksienė R. (2016). Development of an HPLC post-column antioxidant assay for *Solidago canadensis* radical scavengers. Nat. Prod. Res..

[39] Xuan J.-J., Balakrishnan P., Oh D.H., Yeo W.H., Park S.M., Yong C.S., Choi H.-G. (2010). Rheological characterization and in vivo evaluation of thermosensitive poloxamer-based hydrogel for intramuscular injection of piroxicam. Int. J. Pharm..

[40] Zhao Z., Gao S., Li Y., Wu F., Shen C. (2021). Gelation of Konjac glucomannan crosslinked by organotitanium chelated with different ligands. J. Sol-Gel Sci. Technol..

[41] Yim S.-H., Nam S.-H. (2016). Physiochemical, nutritional and functional characterization of 10 different pear cultivars (*Pyrus* spp.). J. Appl. Bot. Food Qual..

[42] Simpson M.G., Simpson M.G. (2010). 8—Diversity and Classification of Flowering Plants: Eudicots. Plant Systematics.

[43] Song Y., Tian X., Wang X., Feng H. (2019). Vascular protection of salicin on IL-1β-induced endothelial inflammatory response and damages in retinal endothelial cells. Artif. Cells Nanomed. Biotechnol..

[44] Vlachojannis J.E., Cameron M., Chrubasik S. (2009). A systematic review on the effectiveness of willow bark for musculoskeletal pain. Phytother. Res..

[45] Chrubasik S., Fiebich B., Black A., Pollak S. (2002). Treating low back pain with a Salix extract that inhibits COX 2 and the release of cytokines. Eur. J. Anaesthesiol..

[46] Vlachojannis J., Magora F., Chrubasik S. (2011). Willow Species and Aspirin: Different Mechanism of Actions. Phytother. Res..

[47] Mahdi J.G. (2010). Medicinal potential of willow: A chemical perspective of aspirin discovery. J. Saudi Chem. Soc..

[48] Wölfle U., Haarhaus B., Kersten A., Fiebich B., Hug M.J., Schempp C.M. (2015). Salicin from Willow Bark can Modulate Neurite Outgrowth in Human Neuroblastoma SH-SY5Y Cells. Phytother. Res..

[49] Wroblewska K., Kucinska M., Murias M., Lulek J. (2015). Characterization of new eye drops with choline salicylate and assessment of their irritancy by in vitro short time exposure tests. Saudi Pharm. J..

[50] Wróblewska K.B., Plewa S., Długaszewska J., Froelich A., Muszalska-Kolos I. (2021). Design and evaluation of pharmaceutical availability, stability and quality of modified viscosity eye drops with choline salicylate. Eur. J. Pharm. Sci..

[51] Yan H., Guo Y., Zhang J., Ding Z., Ha W., Harding J.J. (2008). Effect of Carnosine, Aminoguanidine, and Aspirin Drops on the Prevention of Cataracts in Diabetic Rats. Mol. Vis..

[52] Librando A., Carlesimo S.C., Albanese G., Albanese G.M., Migliorini R., Pacella E. (2018). Effectiveness of 0.1% topical salicylic acid on blepharoconjunctivitis affecting glaucoma patients treated with topical prostaglandin analogues: A prospective randomized trial. Int. J. Ophthalmol..

[53] Stanciauskaite M., Marksa M., Babickaite L., Majiene D., Ramanauskiene K. (2021). Comparison of Ethanolic and Aqueous *Populus balsamifera* L. Bud Extracts by Different Extraction Methods: Chemical Composition, Antioxidant and Antibacterial Activities. Pharmaceuticals.

[54] Almuhayawi M.S. (2020). Propolis as a novel antibacterial agent. Saudi J. Biol. Sci..

[55] Przybyłek I., Karpiński T.M. (2019). Antibacterial Properties of Propolis. Molecules.

[56] Nichitoi M.M., Josceanu A.M., Isopescu R.D., Isopencu G.O., Geana E.-I., Ciucure C.T., Lavric V. (2021). Polyphenolics profile effects upon the antioxidant and antimicrobial activity of propolis extracts. Sci. Rep..

[57] Larrosa M., Lodovici M., Morbidelli L., Dolara P. (2008). Hydrocaffeic and *p*-coumaric acids, natural phenolic compounds, inhibit UV-B damage in WKD human conjunctival cells in vitro and rabbit eye in vivo. Free Radic. Res..

[58] Ahmad A., Ahsan H. (2020). Biomarkers of inflammation and oxidative stress in ophthalmic disorders. J. Immunoass. Immunochem..

[59] Lodovici M., Caldini S., Morbidelli L., Akpan V., Ziche M., Dolara P. (2009). Protective effect of 4-coumaric acid from UVB ray damage in the rabbit eye. Toxicology.

[60] Saccà S.C., Roszkowska A.M., Izzotti A. (2013). Environmental light and endogenous antioxidants as the main determinants of non-cancer ocular diseases. Mutat. Res. Mutat. Res..

[61] Sudha P.N., Rose M.H., Kim S.-K. (2014). Beneficial Effects of Hyaluronic Acid. Marine Carbohydrates: Fundamentals and Applications, Part A.

[62] Ke C., Sun L., Qiao D., Wang D., Zeng X. (2011). Antioxidant acitivity of low molecular weight hyaluronic acid. Food Chem. Toxicol..

[63] Zheng Q., Li L., Liu M., Huang B., Zhang N., Mehmood R., Nan K., Li Q., Chen W., Lin S. (2020). In situ scavenging of mitochondrial ROS by anti-oxidative MitoQ/hyaluronic acid nanoparticles for environment-induced dry eye disease therapy. Chem. Eng. J..

[64] Juncan A., Moisă D., Santini A., Morgovan C., Rus L.-L., Vonica-Țincu A., Loghin F. (2021). Advantages of Hyaluronic Acid and Its Combination with Other Bioactive Ingredients in Cosmeceuticals. Molecules.

[65] Beaussart A., Retourney C., Quilès F., Morais R.D.S., Gaiani C., Fiérobe H.-P., El-Kirat-Chatel S. (2020). Supported lysozyme for improved antimicrobial surface protection. J. Colloid Interface Sci..

[66] Dekina S.S., Romanovskaya I.I., Ovsepyan A.M., Balashova M.V. (2015). Sterilization of Ocular Medical Inserts with Immobilized Proteins. Pharm. Chem. J..

[67] Jumelle C., Gholizadeh S., Annabi N., Dana R. (2020). Advances and limitations of drug delivery systems formulated as eye drops. J. Control. Release.

[68] Beard M.C., Cobb L., Grant C., Varadarajan A., Henry T., Swanson E.A., Kundu S., Priddy L.B. (2020). Autoclaving of Poloxamer 407 hydrogel and its use as a drug delivery vehicle. J. Biomed. Mater. Res. Part B Appl. Biomater..

[69] Mandal S., Prabhushankar G., Thimmasetty M.K., Geetha M. (2012). Formulation and evaluation of an in situ gel-forming ophthalmic formulation of moxifloxacin hydrochloride. Int. J. Pharm. Investig..

[70] Kesavan K., Kant S., Pandit J.K. (2016). Therapeutic Effectiveness in the Treatment of Experimental Bacterial Keratitis with Ion-activated Mucoadhesive Hydrogel. Ocul. Immunol. Inflamm..

[71] Rossatto A., dos Santos J.T., Arlindo M.Z.F., de Morais M.S., de Souza T.D., Ogrodowski C.S. (2022). Hyaluronic acid production and purification techniques: A review. Prep. Biochem. Biotechnol..

[72] Szabó A., Szabó B., Balogh E., Zelkó R., Antal I. (2013). Structural elucidation of hyaluronic acid gels after heat sterilisation. Polym. Test..

[73] Kuo J., Vladimir P. (1997). Steam-Sterilizing Solid Hyaluronic Acid. U.S. Patent.

[74] Russo E., Villa C. (2019). Poloxamer Hydrogels for Biomedical Applications. Pharmaceutics.

[75] Gupta B., Mishra V., Gharat S., Momin M., Omri A. (2021). Cellulosic Polymers for Enhancing Drug Bioavailability in Ocular Drug Delivery Systems. Pharmaceuticals.

[76] Barse R., Kokare C., Tagalpallewar A. (2016). Influence of hydroxypropylmethylcellulose and poloxamer composite on developed ophthalmic in situ gel: Ex vivo and in vivo characterization. J. Drug Deliv. Sci. Technol..

[77] Rahman M.Q., Chuah K.-S., A Macdonald E.C., Trusler J.P.M., Ramaesh K. (2012). The effect of pH, dilution, and temperature on the viscosity of ocular lubricants—shift in rheological parameters and potential clinical significance. Eye.

[78] Mahboobian M.M., Mohammadi M., Mansouri Z. (2020). Development of thermosensitive in situ gel nanoemulsions for ocular delivery of acyclovir. J. Drug Deliv. Sci. Technol..

[79] Sun J., Zhou Z. (2018). A novel ocular delivery of brinzolamide based on gellan gum: In vitro and in vivo evaluation. Drug Des. Dev. Ther..

[80] Asasutjarit R., Thanasanchokpibull S., Fuongfuchat A., Veeranondha S. (2011). Optimization and evaluation of thermoresponsive diclofenac sodium ophthalmic in situ gels. Int. J. Pharm..

[81] Irimia T., Dinu-Pîrvu C.-E., Ghica M.V., Lupuleasa D., Muntean D.-L., Udeanu D.I., Popa L. (2018). Chitosan-Based In Situ Gels for Ocular Delivery of Therapeutics: A State-of-the-Art Review. Mar. Drugs.

[82] Lim L.T., Ah-Kee E.Y., Collins C.E. (2014). Common eye drops and their implications for pH measurements in the management of chemical eye injuries. Int. J. Ophthalmol..

[83] Tsai C.-H., Wang P.-Y., Lin I.-C., Huang H., Liu G.-S., Tseng C.-L. (2018). Ocular Drug Delivery: Role of Degradable Polymeric Nanocarriers for Ophthalmic Application. Int. J. Mol. Sci..

[84] Achouri D., Alhanout K., Piccerelle P., Andrieu V. (2013). Recent advances in ocular drug delivery. Drug Dev. Ind. Pharm..

[85] Seah I., Loh X.J., Su X. (2020). A topical gel for extended ocular drug release. Nat. Biomed. Eng..

[86] Wei Y., Li C., Zhu Q., Zhang X., Guan J., Mao S. (2020). Comparison of thermosensitive in situ gels and drug-resin complex for ocular drug delivery: In vitro drug release and in vivo tissue distribution. Int. J. Pharm..

[87] Deshkar S.S., Palve V.K. (2019). Formulation and development of thermosensitive cyclodextrin-based in situ gel of voriconazole for vaginal delivery. J. Drug Deliv. Sci. Technol..

[88] Patel A., Cholkar K., Agrahari V., Mitra A.K. (2013). Ocular drug delivery systems: An overview. World J. Pharmacol..

[89] Kuno N., Fujii S. (2011). Recent Advances in Ocular Drug Delivery Systems. Polymers.

[90] Austermann H., Schaeffel F., Mathis U., Hund V., Mußhoff F., Ziemssen F., Schnichels S. (2021). Corneal Penetration of Low-Dose Atropine Eye Drops. J. Clin. Med..

