# Correction: Stanciauskaite et al. Balsam Poplar Buds: Extraction of Potential Phenolic Compounds with Polyethylene Glycol Aqueous Solution, Thermal Sterilization of Extracts and Challenges to Their Application in Topical Ocular Formulations. *Antioxidants* 2022, *11*, 1771

**DOI:** 10.3390/antiox11122320

**Published:** 2022-11-23

**Authors:** Monika Stanciauskaite, Mindaugas Marksa, Liudas Ivanauskas, Kristina Ramanauskiene

**Affiliations:** 1 Department of Clinical Pharmacy, Faculty of Pharmacy, Lithuanian University of Health Sciences, Sukileliai Avenue 13, LT-50162 Kaunas, Lithuania; 2 Department of Drug Chemistry, Faculty of Pharmacy, Lithuanian University of Health Sciences, Sukileliai Avenue 13, LT-50162 Kaunas, Lithuania; 3 Department Analytical & Toxicological Chemistry, Faculty of Pharmacy, Lithuanian University of Health Sciences, Sukileliai Avenue 13, LT-50162 Kaunas, Lithuania

In the original publication [1], there was a mistake in Table 4 as published; a technical error with table overlapping. The active compounds column was duplicated, and the pH value overlapped with the viscosity results from part B of Table 3. The correct Table 4 appears below. The authors state that the scientific conclusions are unaffected. This correction was approved by the Academic Editor. The original publication has also been updated.

## Figures and Tables

**Table 4:** Physicochemical properties of ophthalmic gels formulations after 30 days (mean, SD, *n* = 3).

	pH	SD	Vsc, mPa·s 21 ± 1 °C	SD	Active Compounds %	SD	Appearance
BH12	6.54	0.08	30.93	3.15	98.95	4.07	Clear/yellowish
BC12	6.57	0.06	29.42	2.47	98.75	2.94	Clear/yellowish
BHA12	6.53	0.07	29.71	5.1	97.68	4.88	Clear/yellowish
BH8	6.5	0.07	13.36	2.93	98.36	6.81	Clear/yellowish
BC8	6.5	0.04	12.96	1.46	99.2	2.21	Clear/yellowish
BHA8	6.49	0.08	14.4	3.96	98.81	3.46	Clear/yellowish
BH10	6.48	0.04	18.17	3.68	97.19	4.17	Clear/yellowish
BC10	6.49	0.08	17.5	2.48	96.94	4.87	Clear/yellowish
BHA10	6.48	0.09	18.31	4.49	98.34	2.88	Clear/yellowish
BH15	6.6	0.08	61.33	6.14	97.53	5.69	Clear/yellowish
BC15	6.67	0.07	60.52	4.34	99.13	4.15	Clear/yellowish
BHA15	6.68	0.07	54.38	2.76	98.56	3.75	Clear/yellowish
